# Qijiao Shengbai capsule ameliorates ulcerative colitis in mice by modulating gut microbiota and protecting the intestinal barrier, associated with suppression of the TLR4/MyD88/NF-κB pathway

**DOI:** 10.3389/fmicb.2026.1875650

**Published:** 2026-09-16

**Authors:** Yi Lu, Min Zhang, Siyue Jiang, Shuo Zhang, Zhiyu Chen, Jueben Wang, Li Liu, Pengjiao Wang, Xiuli Gao

**Affiliations:** 1 State Key Laboratory of Discovery and Utilization of Functional Components in Traditional Chinese Medicine & School of Pharmaceutical Sciences, Guizhou Medical University, Guiyang, China; 2 Engineering Research Center of Microbiology and Biochemical Pharmaceutical, Guizhou Provincial Department of Education, Guiyang, China; 3 Experimental Animal Center of Guizhou Medical University, Guiyang, China; 4 Guizhou Hanfang Pharmaceutical Co., Ltd., Guiyang, China; 5 Guizhou Provincial Engineering Research Center of Food Nutrition and Health, Guizhou Medical University, Guiyang, China

**Keywords:** gut microbiota, intestinal barrier, Qijiao Shengbai capsule, TLR4/MyD88/NF-κB pathway, ulcerative colitis

## Abstract

**Background:**

Qijiao Shengbai capsule (QJSB) is a classic traditional Chinese compound formulation composed of seven herbal ingredients, primarily used clinically to treat leukopenia. Individual herbs and their active components within QJSB demonstrate favorable efficacy against ulcerative colitis (UC). Nevertheless, no evidence exists of QJSB being a full-form prescription for treating UC.

**Methods:**

This study identified the chemical composition of QJSB using UHPLC-ESI-Q-Exactive Plus Orbitrap-MS. At the same time, a mouse model of ulcerative colitis (UC) was established using DSS to examine the effects of QJSB on disease symptoms, inflammatory factors, intestinal barrier function, and colonic histopathological changes. Furthermore, metabolomics, 16S rRNA sequencing, and network pharmacology were combined to analyze the potential mechanisms underlying the effects of QJSB intervention in UC-affected mice, and key pathways predicted by network pharmacology were validated using Western blot analysis.

**Results:**

The results showed that QJSB treatment significantly alleviated DSS-induced colitis symptoms in UC mice, as evidenced by weight recovery, a decrease in the disease activity index (DAI), improvement in colonic shortening, and reduced tissue damage. Gut microbiota analysis indicated that QJSB modulates the composition of the gut microbiota by upregulating the abundance of *Akkermansia* and *Lactobacillus* and downregulating the abundance of *Bacteroides*. Metabolomics results suggest that QJSB may help correct metabolic disturbances involving amino acids, linoleic acid, arachidonic acid, and alpha-linolenic acid. Furthermore, QJSB was associated with reduced levels of pro-inflammatory factors (TNF-α, IL-6, IL-1β), lipopolysaccharide (LPS), and myeloperoxidase (MPO), as well as improved intestinal barrier function. Western blot results suggest that QJSB intervention may be associated with the suppression of TLR4/MyD88/NF-κB signaling pathway activation.

**Conclusion:**

The present findings suggest that QJSB may exert therapeutic effects against UC, potentially through mechanisms involving the inhibition of inflammatory responses, protection of intestinal barrier function, regulation of metabolic homeostasis, and restoration of gut microbial ecology. These results imply that QJSB could serve as a potential therapeutic candidate for UC and provide a new basis for its clinical application.

## 1 Introduction

Ulcerative colitis (UC), a chronic and nonspecific form of inflammatory bowel disease (IBD), has as its core feature continuous and diffuse inflammation predominantly localized to the colon and rectum (Kobayashi et al., 2020; Feuerstein et al., 2019). Its common clinical manifestations include persistent or recurrent diarrhea, bloody stools, and abdominal pain, which seriously reduce the life quality of patients and increase the risk of colorectal cancer (Feuerstein et al., 2019). UC incidence and prevalence rates have risen rapidly worldwide in recent years, especially in younger people, making it a key public health concern (Kobayashi et al., 2020). The existing first-line UC clinical therapies are 5-aminosalicylates, immunosuppressants, and glucocorticoids (Jeong et al., 2019; Sands et al., 2019; Rubin et al., 2017). Although these drugs demonstrate efficacy, their side effects—including gastrointestinal reactions, hepatic and renal impairment, and bone marrow suppression—severely limit clinical application (Burri et al., 2020; Danese et al., 2020; Digby-Bell et al., 2020). Therefore, identifying effective alternative therapies for UC holds significant clinical importance.

UC etiology is multifactorial and complex, arising from the interplay between genetic predisposition, impaired intestinal barrier function, metabolic disturbances, and gut microbiota imbalance (Le Berre et al., 2023; Hyun and Cheon, 2025; Nishida et al., 2018). Recent studies indicate that dysbiosis of intestinal microbes has been approved as an important cause of UC development and progression (Zhu et al., 2022). Notably, the profile of the intestinal microbiota in UC patients exhibits marked abnormalities, characterized by reduced microbial diversity alongside enrichment of potentially pathogenic bacteria, including *Bacteroides* and *Proteobacteria* (Lin et al., 2024; Qu et al., 2021). These shifts in the gut microbial community, alongside disruptions to metabolic equilibrium, lead to compromised function of the intestinal mucosal barrier. Furthermore, lipopolysaccharides (LPS) released by pathogenic bacteria such as *Bacteroides* exacerbate structural damage and inflammatory responses in the intestinal barrier (Mancuso et al., 2005; Qiu et al., 2014), thereby promoting the progression of UC.

The TLR4/MyD88/NF-κB signaling cascade is a key regulator of UC progression (Chen et al., 2022), and its abnormal activation further exacerbates intestinal epithelial barrier dysfunction. Disrupted gut microbiota composition and metabolic homeostasis imbalance lead to increased abundance of pathogenic bacteria (such as *Bacteroides*), thereby elevating LPS levels (Martini et al., 2017). As a ligand for Toll-like receptor 4 (TLR4), LPS triggers TLR4 and activates the downstream MyD88/NF-κB pathway, ultimately resulting in upregulated gene expression of abundant pro-inflammatory cytokines, thus exacerbating intestinal inflammation (Xiong et al., 2022).

Given its multi-target efficacy, favorable safety profile, and cost-effectiveness, Traditional Chinese Medicine (TCM) is increasingly regarded as a valuable complementary and alternative therapeutic option for managing UC (Liu et al., 2022; Xu et al., 2025). In recent years, a substantial body of research has confirmed that TCM formulas can suppress NF-κB-mediated inflammatory responses, thereby reducing pro-inflammatory cytokine levels and alleviating colonic tissue damage, suggesting that the NF-κB pathway may represent a pivotal node through which TCM exerts its anti-UC effects (Shi et al., 2025; Dai et al., 2023). Qijiao Shengbai capsule (QJSB) is a classic Miao ethnic formula composed of seven herbs—*Astragali radix* (Huang Qi), *Indigofera stachyoides* Lindl. (Xue Renshen), *Equus asinus L*. (EJiao), *Angelica sinensis* (Oliv.), Diels (Dang Gui), *Ziziphus jujuba* Mill. (Da Zao), *Epimedium brevicornu* Maxim. (Yin Yanghuo), and *Sophora flavescens* Ait. (Ku Shen)—blended in specific proportions. It has been clinically applied to treat leukopenia (Jiang et al., 2024). Our prior research demonstrated that QJSB significantly modulates gut microbiota and enhances intestinal immunity (Cao et al., 2023). Furthermore, individual herbs and active components within the QJSB formulation have exhibited clear anti-UC efficacy (Liu et al., 2023; Zhao et al., 2025; Liu et al., 2024; Zhu et al., 2025), and multiple constituents have been verified to alleviate intestinal inflammation through intervention in the NF-κB signaling pathway (Peng et al., 2020; Li et al., 2022). Therefore, we hypothesize that QJSB may serve as a holistic compound for treating UC.

In this context, the present investigation seeks to clarify the protective mechanisms and efficacy of QJSB capsules in a mouse model of UC. By utilizing dextran sulfate sodium (DSS) to induce colitis, an integrated approach combining network pharmacology, metabolomic profiling, and 16S ribosomal RNA sequencing was applied. This comprehensive strategy demonstrated that QJSB treatment mitigates inflammatory processes, restores intestinal barrier integrity, corrects metabolic dysregulation, and rebalances gut microbiota composition. The findings offer a scientific foundation for the further development of QJSB and present a potential new treatment avenue for individuals with UC.

## 2 Materials and methods

### 2.1 Materials and reagents

QJSB samples (Lot Number: Z20025027) were obtained from Guizhou Hanfang Pharmaceutical Co., Ltd. (Guiyang, China). 5-ASA (Cat. No. HY-15027) was purchased from Yisai Pharmaceutical Co., Ltd. (Shanghai, China). Dextran sulfate sodium (DSS, MW: 40,000 Da, Cat. No. A523162) was purchased from AmBeed (USA). The IL-6 (Cat. No. RXW203049M), TNF-α (Cat. No. RXW202412M), and IL-1β (Cat. No. RXW203063M) enzyme-linked immunosorbent assay (ELISA) kits were obtained from Ruixin Biotechnology Co., Ltd. (Quanzhou, China), while the MPO (Cat. No. A044-1-1) assay kit was purchased from Nanjing Jiancheng Bioengineering Institute (Nanjing, China). The LPS (Cat. No. F30715-A) ELISA kit was purchased from FANAKEW (Shanghai, China). ZO-1, occludin, claudin-1, and GAPDH antibodies were all obtained from Hua'an Biological Technology Co., Ltd. (Hangzhou, China). TLR4, MyD88, IκBα, p-IκBα, NF-κB p65, NF-κB p-p65, iNOS, and COX-2 antibodies were obtained from Proteintech (Wuhan, China). All reagents were of analytical grade except for UPLC-grade methanol, acetonitrile, and formic acid (Merck, Germany).

### 2.2 QJSB sample preparation and component identification

QJSB powder (300 mg) was mixed with 3,000 μL of 80 % methanol aqueous solution and grinding beads. The mixture was homogenized using a grinder for 5 min, followed by vortexing for 10 min. After centrifugation at 12,000 rpm for 10 min at 4 °C, the supernatant was filtered and subjected to UHPLC-ESI-Q-Exactive Plus Orbitrap-MS analysis (Jiang et al., 2024).

To preliminarily identify potential active components in QJSB, a Waters (USA) HSS T3 UPLC column (2.1 × 100 mm, 1.8 μm) was used. The mobile phase consisted of acetonitrile (A) and a 0.1 % aqueous formic acid solution (B). The injection volume, flow rate, and column temperature were set to 2 μL, 0.3 mL/min, and 40 °C, respectively. The gradient elution program was as follows: 0–2 min, 5 %−5 % A; 2–42 min, 5 %−95 % A; 42–47 min, 95 %−95 % A; 47–47.1 min, 95 %−5 % A; 47.1–50 min, 5 %−5 % A. Subsequently, a Thermo Scientific Q Exactive Plus mass spectrometer (Thermo Fisher Scientific, USA) was used to simultaneously scan positive and negative ions via full-scan/data-dependent MS^2^ (dd-MS^2^). The capillary voltage was set to 3.5 kV in positive ion mode and 2.5 kV in negative ion mode. The HESI-II source temperature was 350 °C, and the capillary temperature was 320 °C. The sheath gas flow rate was 35 arb, and the auxiliary gas flow rate was 10 arb. The scan range was m/z 100–1500, with an MS^1^ resolution of 70,000 and an MS/MS resolution of 17,500. The normalized collision energies (NCE) were set to 20, 40, and 60 eV. The raw data were imported into Compound Discoverer 3.3 software, and the relative mass tolerance was set to less than 5 ppm. Based on accurate mass and MS^1^ and MS^2^ fragmentation information, data analysis was performed using Xcalibur software, and the results were compared with the mzCloud, mzVault, and ChemSpider databases, as well as with data reported in the literature.

### 2.3 Animals and grouping

Male BALB/c mice (SPF grade, aged 6–8 weeks) were obtained from the Experimental Animal Center of Guizhou Medical University [License No. SCXK (Xiang): 2024-0021; supplied by Changsha Tianqin Biotechnology Co., Ltd., China]. All mice were maintained under defined housing conditions (24 ± 2 °C, 60 ± 5 % relative humidity) with a 12-h light/dark cycle, provided with free access to food and water, and subjected to a one-week acclimation period prior to experimentation. All experimental procedures complied with the ethical requirements of the Animal Ethics Committee of Guizhou Medical University [License No. SYXK (Gui) 2023–0002]. Mice were weighed and randomly divided into six groups (*n* = 8/group): (1) Control group; (2) Model group; (3) QJSB-L group (DSS+QJSB 0.5 g/kg/d); (4) QJSB-M group (DSS+QJSB 1 g/kg/d); (5) QJSB-H group (DSS+QJSB 2 g/kg/d); (6) 5-aminosalicylic acid (5-ASA 0.1 g/kg/d) positive drug group (Wang et al., 2024). Except for the normal control group, which had unrestricted access to purified water throughout the study, all other groups had unrestricted access to 3.5 % (w/v) DSS solution. The DSS solution was replaced daily to maintain the 7-day colitis induction period. From day 4 of modeling until the end of the experiment (day 10), each drug intervention group was administered the corresponding dose of the drug solution via daily oral gavage. During the experiment, body weight, fecal consistency, and fecal occult blood were recorded daily for each mouse. The disease activity index (DAI) was calculated as the average of three subscores evaluating weight loss, stool consistency, and rectal bleeding. Detailed scoring criteria are provided in Supplementary Table 1 (Hassan and Hassan, 2019). Blood samples, colon tissue, and cecal contents were collected from the mice on day 11 of the experiment and kept at −80 °C for later examination. Colon length was measured by an investigator who was blinded to the group allocation.

### 2.4 Measurement of MPO, LPS, and cytokine levels

Levels of TNF-α, IL-6, and IL-1β in serum and colon tissue, along with MPO in colon tissue and LPS in feces, were measured using ELISA kits strictly following the manufacturers' instructions.

### 2.5 Immunofluorescence staining

Colon sections were initially equilibrated at room temperature (20 °C) for 30 min within an incubator, followed by three rinses using phosphate-buffered saline (PBS). Subsequently, they were placed in EDTA antigen retrieval solution and subjected to antigen retrieval at 95 °C in a water bath for 10 min, followed by natural cooling to room temperature. Thereafter, a blocking step was performed using 5 % bovine serum albumin for one h at ambient temperature. The sections were then incubated with specific primary antibodies—directed against phosphorylated IκBα (1:200), phosphorylated p65 (1:200), ZO-1 (1:200), Claudin-1 (1:200), and Occludin (1:50)—in a humidified environment at 4 °C overnight. The next day, after rewarming, species-specific fluorescent secondary antibodies were added. The slides were then subjected to a 2 h incubation at 37 °C under light-protected conditions. Subsequently, cell nuclei were counterstained with DAPI for five min before final mounting. Fluorescence images were captured under an inverted fluorescence microscope. Three non-overlapping fields of view were randomly selected per section, and the mean fluorescence intensity was quantified using ImageJ software to assess changes in intestinal epithelial barrier-related proteins.

### 2.6 Colon histopathology examination

Colon tissue specimens were preserved by immersion in a 4 % paraformaldehyde solution for a period exceeding 24 h. They were then cleared with xylene, impregnated with paraffin, and dehydrated using a graded ethanol series. A Leica EG1150H embedding machine was used for paraffin embedding, and the embedded blocks were kept at 4 °C The paraffin blocks were pre-cooled before sectioning. A Leica RM2550 manual microtome was employed to obtain 4 μm thick sections, which were then placed on anti-shedding slides and baked for two h at 60 °C. Following deparaffinization and rehydration, tissue sections underwent standard hematoxylin and eosin (H&E) staining, were mounted with neutral balsam, and subsequently observed under a light microscope.

Fresh colon samples were fixed in 4 % paraformaldehyde for at least 24 h. After dehydration in a gradient of ethanol, clearing with xylene, and paraffin permeation, the samples were embedded in paraffin using a Leica EG1150H embedding machine and stored at 4 °C. Prior to sectioning, the paraffin blocks were pre-cooled, and 4-μm sections were cut using a Leica RM2550 manual microtome. The sections were mounted on anti-slip slides and baked at 60 °C for 2 h. After routine dewaxing and rinsing with water, the sections were stained with hematoxylin and eosin (H&E) and mounted with neutral resin. The sections were then examined under a light microscope, and pathological changes were recorded. Pathological assessment was performed by an independent pathologist under blinded conditions, quantifying inflammatory cell infiltration (grades 0–3) and tissue damage (grades 0–3) to generate H&E scores for each group. The scoring criteria were as follows: inflammatory cell infiltration: no infiltration, 0 points; occasional inflammatory cells in the lamina propria, 1 point; inflammatory cells extending into the submucosa, 2 points; inflammatory cells extending into the serosa, 3 points. Tissue damage: no mucosal damage, 0 points; presence of lymphoepithelial lesions, 1 point; superficial mucosal erosion or localized ulcers, 2 points; extensive mucosal damage extending to deeper intestinal wall structures, 3 points. The final H&E score was derived from a comprehensive assessment of the severity of inflammatory cell infiltration and tissue damage.

### 2.7 Fecal and colonic metabolomics

Fecal samples (50 mg) were homogenized in 500 μL of pre-chilled acetonitrile–methanol–water (1:2:2, v/v/v) for 2 min, followed by vortexing for 5 min, ultrasonic extraction in an ice bath for 40 min, and centrifugation at 15,000 rpm for 10 min at 4 °C. Colon tissue samples (100 mg) were homogenized under low-temperature conditions and mixed with 500 μL of pre-chilled methanol–acetonitrile–water (1:1:1, v/v/v). After vortexing for 1 min and ultrasonic extraction on ice for 15 min, the homogenates were kept at −20 °C overnight and then centrifuged. The supernatants from all samples were filtered through a 0.22-μm membrane filter and transferred to sample vials for subsequent analysis. In addition, quality control (QC) samples for method validation were prepared by pooling 5-μL aliquots of each fecal or colon tissue supernatant.

Metabolomic analysis was performed using a UHPLC-Q-Exactive Plus Orbitrap-MS system (Thermo Fisher Scientific, USA) equipped with a ZORBAX Eclipse Plus C18 column (2.1 × 100 mm, 1.8 μm; Agilent Technologies, USA). The mobile phase consisted of water (0.1 % formic acid, A) and acetonitrile (0.1 % formic acid, B), with the following gradient elution: 0–2.5 min, 2 % B; 2.5–5 min, 2–40 % B; 5–12 min, 40–100 % B; 12–16 min, 100 % B; 16–16.1 min, 100–2 % B; 16.1–19 min, 2 % B, at a flow rate of 0.3 mL/min. The ion spray voltages were set to 3.5 kV (positive) and 2.8 kV (negative), with a mass scan range of m/z 100–1,500. The sheath gas heater temperature and capillary temperature were maintained at 350 °C and 320 °C, respectively. The dynamic exclusion duration was 3 s, and the S-lens RF level was set to 50. Mass spectrometry data were preprocessed using Compound Discoverer 3.2 software (Thermo Fisher Scientific, USA) for peak identification, alignment, and retention time correction. Principal component analysis (PCA) and orthogonal partial least-squares discriminant analysis (OPLS-DA) were performed using SIMCA-P 14.1 (Umetrics, AB, Sweden), and the OPLS-DA models were validated by 200 permutation tests. Endogenous metabolites were identified by matching the MS^1^ and MS/MS fragmentation information against the mzCloud, mzVault, ChemSpider, KEGG (https://www.genome.jp/kegg/), and HMDB (http://www.hmdb.ca/) databases, with a precursor ion mass tolerance within ±5 ppm. MetaboAnalyst 5.0 was used for metabolic pathway enrichment analysis, and pathways with an impact value greater than 0.1 were considered significantly affected. Differential metabolites were screened using the Benjamini–Hochberg false discovery rate (FDR) method for multiple testing correction, with an adjusted *q*-value < 0.05 considered statistically significant.

### 2.8 Gut microbiota 16S rRNA high-throughput sequencing

Genomic DNA was extracted from cecal contents according to the instructions for the fecal genomic DNA extraction kit; DNA integrity and concentration were assessed via 1 % agarose gel electrophoresis and NanoDrop One analysis; Using the extracted DNA as a template, the 16S rRNA V3–V4 region (~469 bp) was amplified with primers 338F (5'-ACTCCTACGGGAGGCAGCA-3') and 806R (5'-GGACTACHVGGGTWTCTAAT-3') using Premix Taq (TaKaRa) for PCR amplification; After comparing the concentrations of the PCR products using GeneTools software (v4.03.05.0), they were mixed in equal volumes, purified using the E.Z.N.A.^®^ Gel Extraction Kit, and library preparation was performed according to the standard protocol of the NEBNext^®^ Ultra™ DNA Library Prep Kit for Illumina^®^. Bispecific sequencing was conducted on the DNBSEQ PE250 platform, yielding approximately 60,000 raw reads per sample. Raw data were quality-controlled using fastp (v0.14.1, parameters: -W 4 -M 20), primer removal was performed using cutadapt (v1.14), and assembly was carried out using USEARCH (v10.0.240, minimum overlap 16 bp, maximum mismatch 5 bp); UPARSE was used to cluster data into OTUs at 97 % similarity; UCHIME was used to remove chimeras; after samples were thinned to the same sequencing depth, species annotation was performed based on the SILVA v138 database (confidence threshold 0.8), and chloroplast and mitochondrial sequences were removed.

Differences in alpha-diversity indices (Shannon, Chao1, ACE, Simpson, and Berger-Parker) among groups were analyzed using the Kruskal-Wallis rank-sum test, followed by Dunn's *post-hoc* test for pairwise comparisons. β-diversity was visualized using principal coordinate analysis (PCoA) based on the Bray-Curtis distance matrix. Differentially abundant species were identified using linear discriminant analysis effect size (LEfSe, LDA > 3, *P* < 0.05).

### 2.9 Network pharmacology

The active compounds were identified based on the results of the chemical composition analysis of QJSB and the literature (Jiang et al., 2024). Structural information on the compounds in QJSB was obtained from the PubChem database (https://pubchem.ncbi.nlm.nih.gov/). Potential targets for the compounds were identified by searching SwissTargetPrediction (www.swisstargetprediction.ch/) and TCMSP (Traditional Chinese Medicine Systems Pharmacology Database and Analysis Platform, https://tcmsp-e.com/tcmsp.php), followed by merging and deduplicating the results. Meanwhile, UC**-**related targets were retrieved from GeneCards and OMIM databases, and targets with a score above 1 were selected. The intersection of active compound targets and disease targets yielded the common targets of QJSB and UC. Common targets were analyzed for protein**-**protein interaction (PPI) via the STRING online database. Then, Gene Ontology (GO) annotation and Kyoto Encyclopedia of Genes and Genomes (KEGG) pathway enrichment analysis were conducted on key targets (*p* < 0.01) with humans as the background species, and results were visualized. Finally, a multidimensional “drug**-**component**-**intersection gene” network was constructed using Cytoscape 3.10.3 software, in which nodes represented drugs, key active components, and intersection genes, and edges represented their interaction relationships.

### 2.10 Western blot

Colon tissue was homogenized in pre-chilled lysis buffer. The resulting homogenate was centrifuged at 12,000 rpm for 15 min at 4 °C, and the supernatant was collected for total protein extraction. Protein concentration was determined using a BCA protein assay kit according to the manufacturer's instructions. The extracted protein samples were separated by 8 % sodium dodecyl sulfate-polyacrylamide gel electrophoresis (SDS-PAGE) and transferred to PVDF membranes. After blocking with 5 % skim milk for 2 h at room temperature, the membranes were incubated overnight at 4 °C with primary antibodies against ZO-1 (1:1000), occludin (1:1000), claudin-1 (1:2000), TLR4 (1:1000), MyD88 (1:2000), IκBα (1:1000), p-IκBα (1:1000), NF-κB p65 (1:2000), p-p65 (1:2000), iNOS (1:2000), COX-2 (1:2000), and GAPDH (1:5000). After washing three times with TBST (10 min each), the membranes were incubated with horseradish peroxidase (HRP)-conjugated secondary antibody for 1.5 h at room temperature. The membranes were then washed three times with TBST, and protein bands were visualized using a Tanon 5200 chemiluminescence imaging system. Semi-quantitative analysis of grayscale values was performed using ImageJ software (NIH, Bethesda, MD, USA).

### 2.11 Statistical analysis

Statistical analysis was performed using SPSS 27.0.1 software (IBM Corp., Armonk, NY, USA), and data are presented as mean ± standard error of the mean (SEM). Comparisons between two groups were performed using Student's *t*-test, while comparisons among multiple groups were performed using one-way ANOVA, followed by Tukey's *post hoc* test; nonparametric tests were used for data with unequal variances or non-normal distributions. For repeated-measure data on body weight and the disease activity index (DAI), between-group comparisons were performed using two-way repeated-measures ANOVA. *p* < 0.05 was considered statistically significant.

## 3 Results

### 3.1 Identification of chemical composition of QJSB

The research team had previously conducted a preliminary identification of the chemical constituents of QJSB. To further explore its potential bioactive compounds, we optimized the separation conditions to improve the detection efficiency of these constituents and adjusted the mobile phase elution program for ultra-high-performance liquid chromatography (UPLC). Through these optimizations, this study preliminarily identified 16 chemical constituents that had not been previously identified by the research team (Table 1); the total ion current chromatograms and structures are shown in Figure 1.

**Table 1:** Preliminary identification of chemical constituents in QJSB extract.

NO	Name	Formula	RT [min]	MS1 (M/Z)	MS2 (M/Z)	Source
1	Maackiain	C_16_H_12_O_5_	11.919	285.07538 (−0.31)	181.06483, 137.02335	Xue Renshen
2	Adenine	C_5_H_5_N_5_	1.481	136.06180 (+1.67)	119.03551, 109.05109	Dang Gui
3	Adenosine	C_10_H_13_N_5_O_4_	1.499	268.10388 (−0.45)	136.06178, 85.02880, 57.03419	Dang Gui
4	Apigenin	C_15_H_10_O_5_	14.182	269.04559 (+0.17)	153.01823	Yin Yanghuo
5	Azelaic acid	C_9_H_16_O_4_	12.274	187.09663 (−4.89)	125.09599, 97.06451	EJiao
6	Betaine	C_5_H_11_NO_2_	1.046	118.08646 (+1.72)	59.07361	Huang Qi
7	Choline	C5H13NO	0.995	104.10729 (+3.04)	60.08141, 58.06577	Huang Qi
8	Cycloolivil	C_20_H_24_O_7_	9.326	375.14520 (+0.85)	73.02814, 108.02025, 241.05066, 360.12180	Yin Yanghuo
9	Luteolin-7-O-glucoside	C_21_H_20_O_11_	11.17	447.09336 (+0.8)	284.03192, 285.04083, 329.13998	Ku Shen
10	L-Pyroglutamic Acid	C_5_H_7_NO_3_	1.471	130.05003 (+1.18)	56.05017, 84.04491, 102.05540	Dang Gui
11	Arginine	C_6_H_14_N_4_O_2_	0.931	175.11894 (−0.06)	70.06569, 72.08131, 116.07083, 130.09753	Dang Gui
12	Icaritin	C_21_H_20_O_6_	22.082	369.13333 (+0.18)	107.01318,135.04425, 187.07550, 313.07065	Yin Yanghuo
13	Kaempferol	C_15_H_10_O_6_	11.419	287.05472 (−0.08)	153.01828, 121.02863	Yin Yanghuo
14	Quercetin	C_15_H_10_O_7_	10.914	303.04959 (+0.17)	137.02335, 153.01819, 229.04936	Yin Yanghuo
15	Soyasaponin I	C_48_H_78_O_18_	20.978	941.51257 (+0.66)	143.03387	Huang Qi
16	Sucrose	C_12_H_22_O_11_	1.17	341.10907 (+0.64)	161.04494, 179.05548, 89.02309	Da Zao

**Figure 1:**
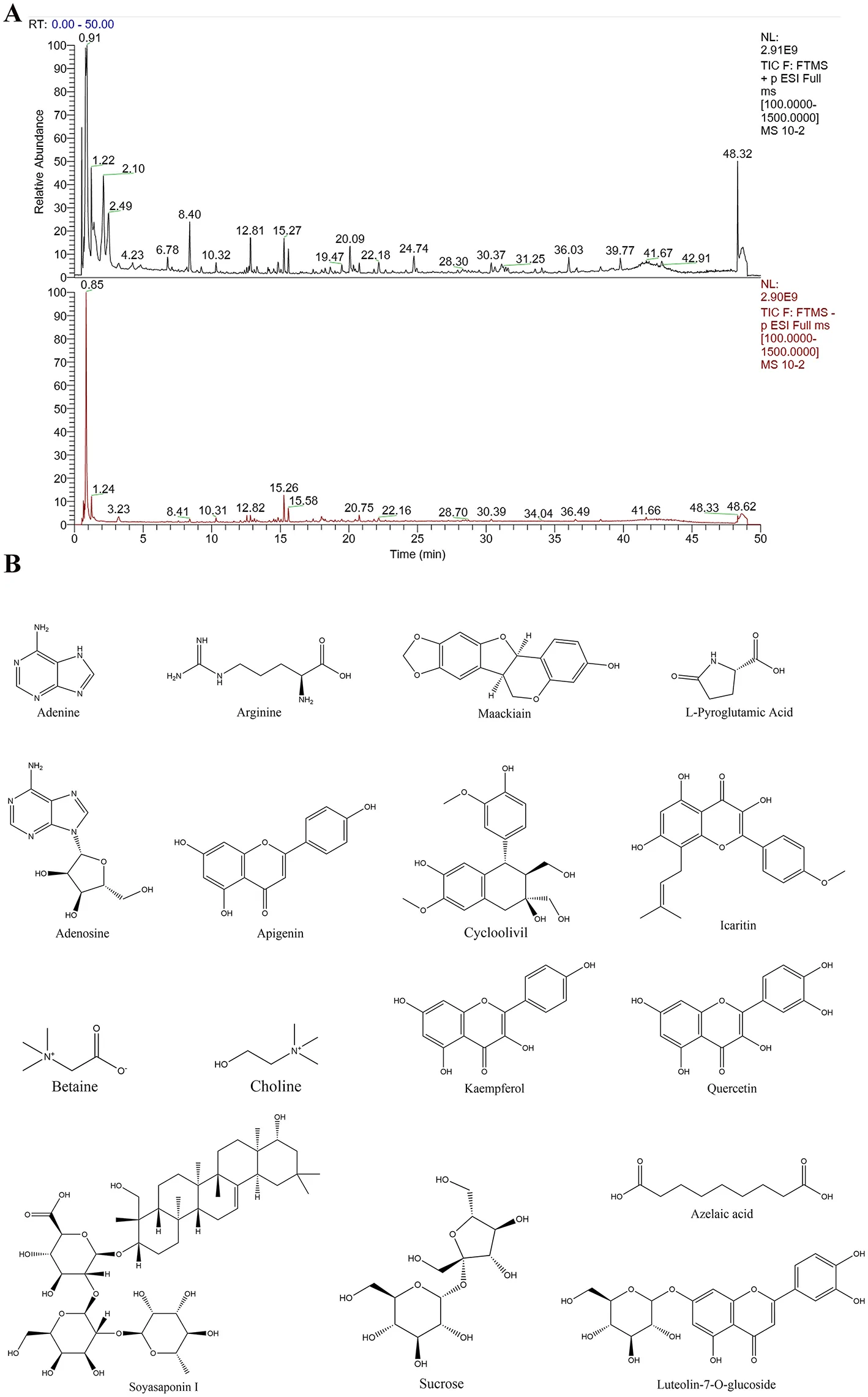
Newly discovered components in QJSB. **(A)** Total ion chromatogram of QJSB; **(B)** Structural formula of QJSB component.

### 3.2 QJSB alleviates disease severity in DSS-induced UC mice

A schematic representation of the ulcerative colitis mouse model establishment is shown in Figure 2A. Relative to the control group, mice in the DSS model group showed weight loss and elevated DAI scores, suggesting that UC model induction was successfully achieved (*p* < 0.05). QJSB significantly improved weight loss and elevated DAI in UC mice (*p* < 0.05). The QJSB-M group demonstrated effects comparable to the 5-ASA group in reversing weight loss and reducing DAI scores (Figures 2B, C). Additionally, we observed a significant reduction in thymus index in the DSS model group (*p* < 0.05), which was alleviated by QJSB and 5-ASA interventions (Figure 2D), suggesting that this drug can mitigate immune organ atrophy associated with ulcerative colitis. DSS-treated mice exhibited a significant reduction in colon length relative to the control group (*p* < 0.05). The QJSB-M and H groups exhibited colon lengths approaching normal levels (*p* < 0.05), demonstrating similar efficacy to the 5-ASA group (Figures 2E, F), while the QJSB-L grou*p* exhibited no significant effect (*p* > 0.05).

**Figure 2:**
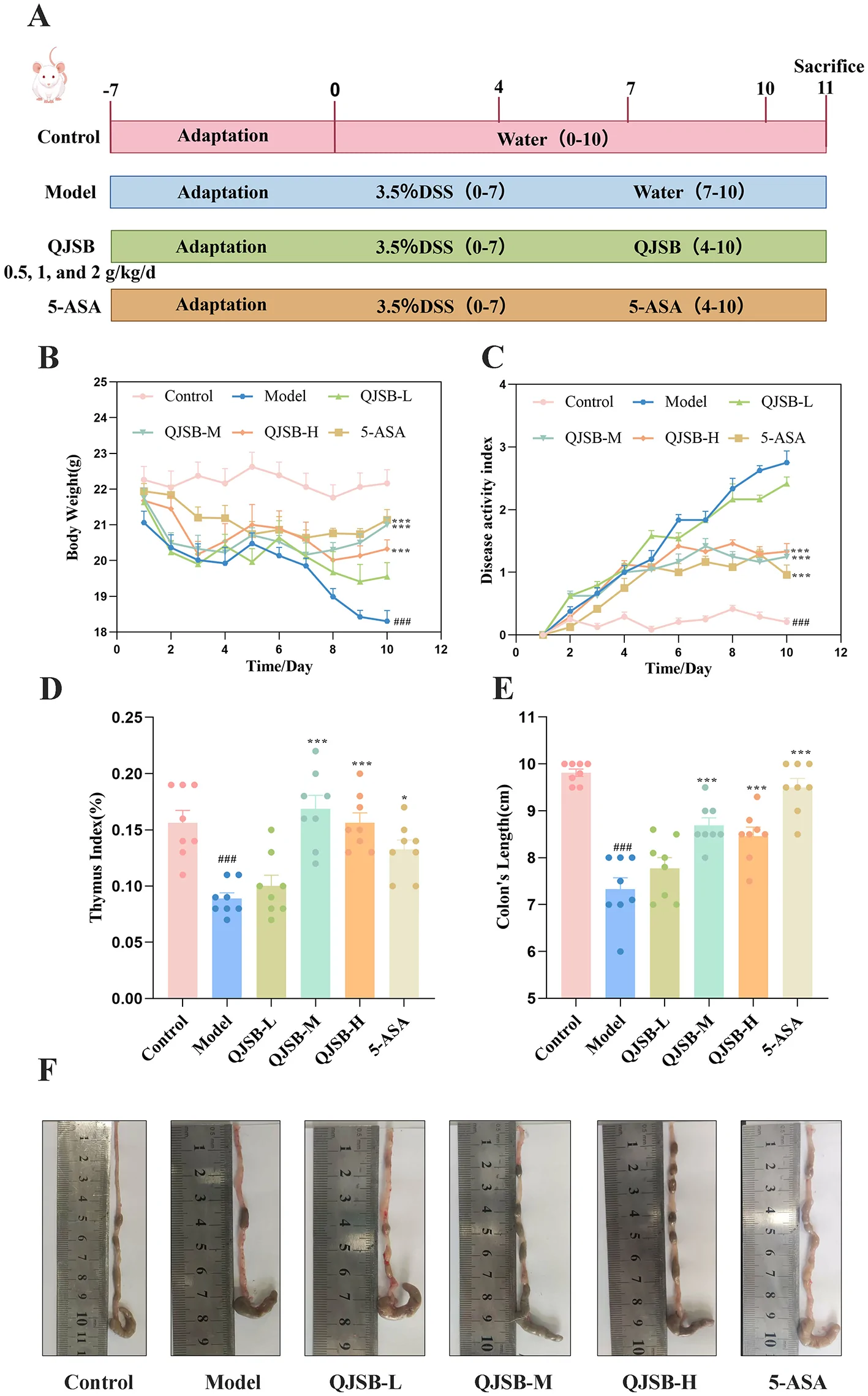
QJSB alleviates the disease severity in DSS-induced UC mice (*n* = 8). **(A)** schematic diagram of animal experiment design; **(B)** mouse body weight; **(C)** disease activity index; **(D)** thymus index ( %); **(E)** colon length; **(F)** representative images of the colon. (###*p* < 0.001 vs. Control; ***p < 0.001, *p < 0.05 vs. Model).

### 3.3 QJSB alleviates DSS-induced gut dysbiosis in UC mice

We examined the effects of QJSB on the gut microbiota of DSS-induced UC mice. Alpha diversity analysis revealed that compared with the control group, the DSS model group exhibited significantly decreased Simpson and Berger-Parker indices, indicating reduced microbial diversity in UC mice. In contrast, QJSB intervention significantly increased the Simpson and Berger-Parker indices (*p* < 0.05, Figures 3A, B). However, in the DSS model group, the Shannon, ACE, and Chao1 indices were markedly elevated, whereas QJSB-treated mice showed a declining trend in these indices, albeit without statistical significance (Supplementary Figure 3). Principal coordinate analysis (PCoA) demonstrated a clear separation between the DSS-induced model group and the QJSB treatment group (Figure 3C); this separation signifies substantial compositional differences in the gut microbial communities of the respective groups. At the family level, compared to the control group, the proportion of Bacteroidaceae was increased in the model group, while QJSB intervention reversed this tendency and enhanced the abundance of Akkermansiaceae (Figure 3D). A comparison at the genus taxonomic rank showed that the relative abundance of *Lactobacillus* was reduced in the DSS model group relative to the control group, whereas the abundance of *Bacteroides* was elevated. QJSB treatment significantly increased the abundance of *Lactobacillus* and *Akkermansia* while decreasing *Bacteroides* abundance (Figures 3E, F). Linear discriminant analysis effect size (LEfSe) analysis indicated that the dominant gut bacteria in the DSS model group were P_Bacteroidota, C_Bacteroidia, and O_Bacteroidales, whereas the dominant bacteria in the QJSB group were f_Akkermansiaceae and *g_Akkermansia*, suggesting these may be key microbial communities mediating QJSB's gut protective effects (Figure 3G).

**Figure 3:**
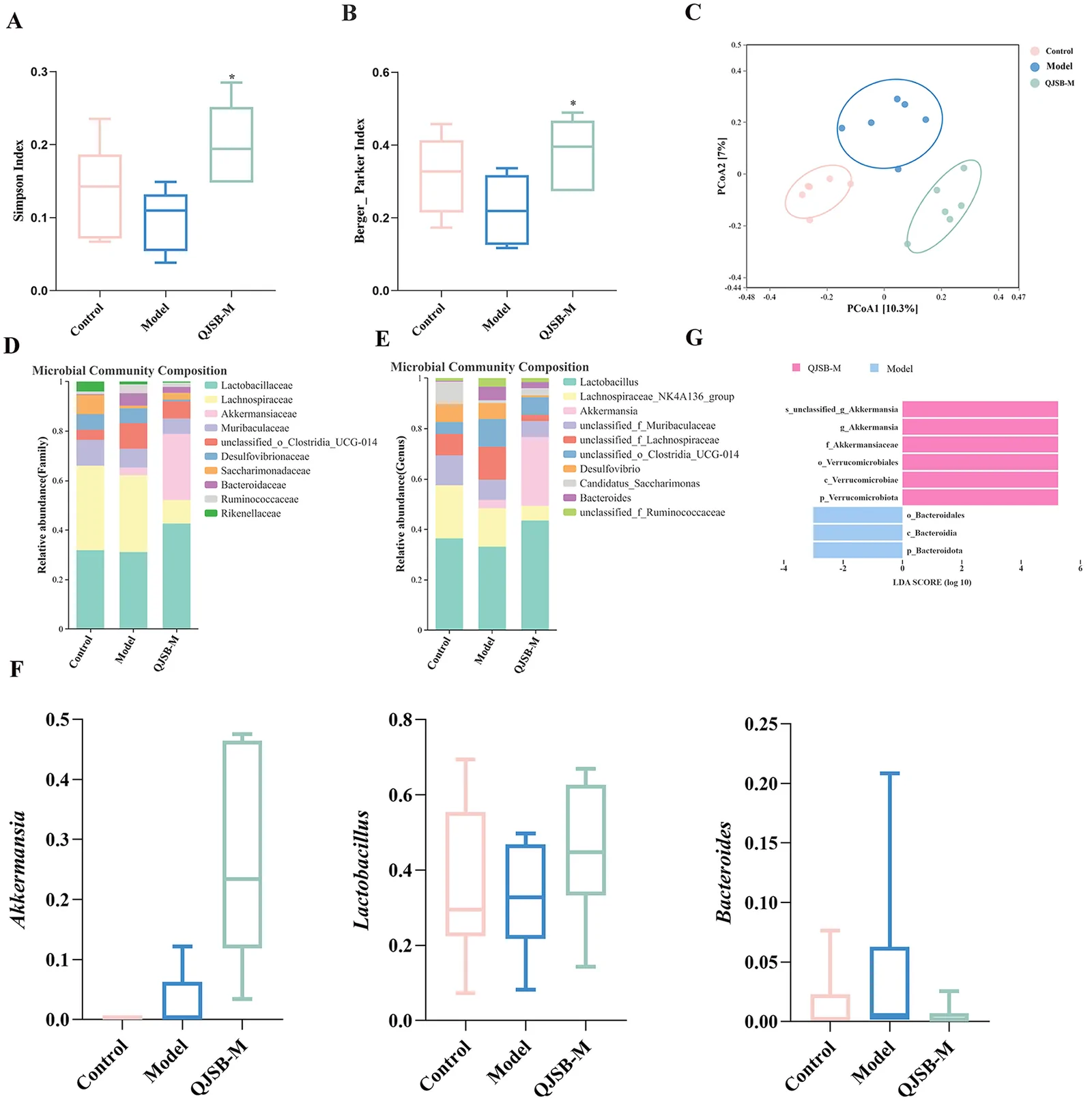
QJSB alleviates DSS-induced gut dysbiosis in UC mice (*n* = 6). **(A)** simpson index; **(B)** berger-parker index; **(C)** PCoA scoring plot; **(D)** The percent of community abundance at the family; **(E)** The percent of community abundance at the genus; **(F)** Relative abundance of key bacterial genera; **(G)** LDA distribution. (*p < 0.05 vs. Model).

### 3.4 Metabolomic analysis of the colon and feces

To investigate the alterations in endogenous metabolites within the colon and feces of UC mice after drug treatment, we conducted a metabolomic analysis of colonic and fecal samples. We utilized principal component analysis (PCA) to understand the trends of separation and differences in metabolic profiles across the groups (Figures 4A, B). The QJSB group demonstrated a clear distinction from the DSS model group and showed a tendency toward the control group, indicating that QJSB might influence the metabolic state of UC mice toward normalcy. The orthogonal partial least squares discriminant analysis (OPLS-DA) model indicated distinct separations among the sample groups (Figures 4C, D). The robustness of the OPLS-DA models was validated by 200-fold permutation tests, which confirmed that the models were reliable and free from overfitting. By applying thresholds of VIP > 1, *P* < 0.05, and fold change (FC) > 1.2 or FC < 0.8, we identified 27 differentially expressed metabolites in the colon and 56 in feces. The heatmap displaying differential metabolites showed 12 upregulated and 15 downregulated metabolites in colon tissue, whereas in fecal samples, 13 metabolites were upregulated and 43 were downregulated (Figures 4E, F). KEGG metabolic pathway enrichment analysis highlighted significant changes in feces, particularly concerning arginine biosynthesis, sphingolipid metabolism, and arginine and proline metabolism, as well as the biosynthesis and metabolism of tyrosine, phenylalanine, and tryptophan, along with arachidonic acid metabolism (Figure 4G). In colon tissue, notable impacts were observed in arachidonic acid metabolism and linoleic acid metabolism (Figure 4H). To investigate whether changes in differential metabolites in feces were associated with key bacterial genera, we performed correlation analyses between differential metabolites and key bacterial genus levels. Results showed that *Akkermansia* exhibited significant negative correlations with metabolites including arachidonic acid, citrulline, and sphingosine while displaying positive correlations with 5-hydroxyindoleacetic acid and ferulic acid. Conversely, *Bacteroides* abundance showed a strong positive correlation with arachidonic acid, alongside a marked negative correlation with 5-hydroxyindoleacetic acid (Figure 4I).

**Figure 4:**
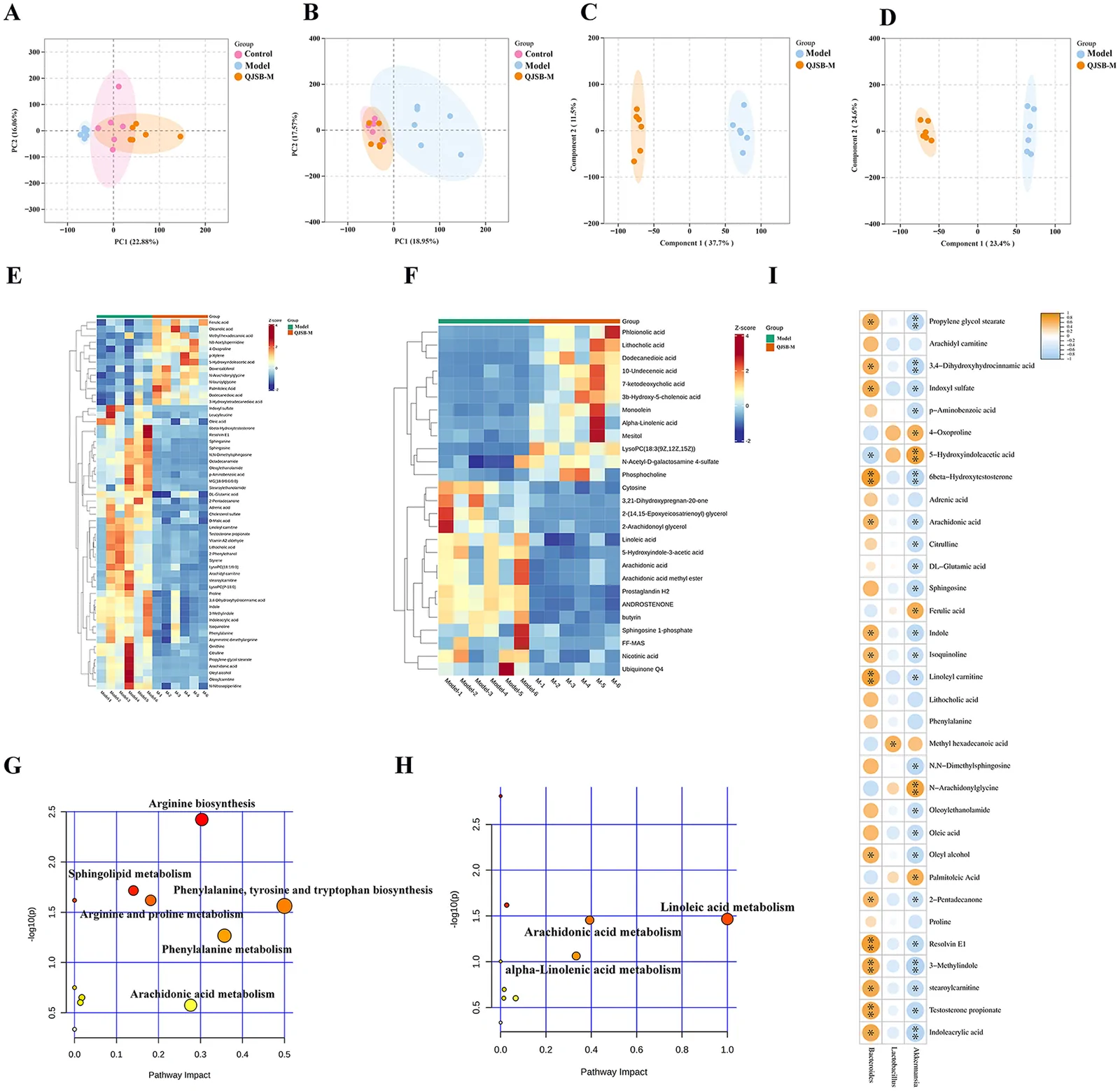
Metabolomics study on the alleviation of UC in mice by QJSB (*n* = 6). **(A)** colon PCA Score chart; **(B)** fecal PCA score plot; **(C)** colon OPLS-DA plot; **(D)** fecal OPLS-DA plot; **(E)** fecal metabolite difference heatmap; **(F)** colon differential metabolite heatmap; **(G)** enrichment bubble chart for fecal metabolic pathways; **(H)** enrichment bubble chart for colon metabolic pathways; **(I)** correlation analysis between key bacterial genera and fecal differential metabolites.

### 3.5 QJSB alleviates intestinal pathological damage in UC mice

Histopathological analysis of colon tissue showed normal overall structure without significant pathology in the control group. In the DSS model group, colon tissue showed extensive ulceration, reduced numbers of intestinal glands and marked inflammatory cell infiltration. Small areas of edema were observed in the submucosa. QJSB intervention effectively ameliorated the intestinal pathological damage, with QJSB-M exhibiting similar effects to 5-ASA (Figures 5A, B). Proteins associated with tight junctions are critical for sustaining intestinal barrier integrity. Immunofluorescence staining revealed marked downregulation of occludin, claudin-1, and ZO-1 in the model group. In contrast, treatment with either QJSB or 5-ASA led to pronounced upregulation of these proteins (*p* < 0.05; Figures 5C–F). Western blot results further validated this trend (*p* < 0.05; Figures 5G–J). These results demonstrate that QJSB effectively relieves pathological injury and enhances the expression of intestinal tight junction proteins.

**Figure 5:**
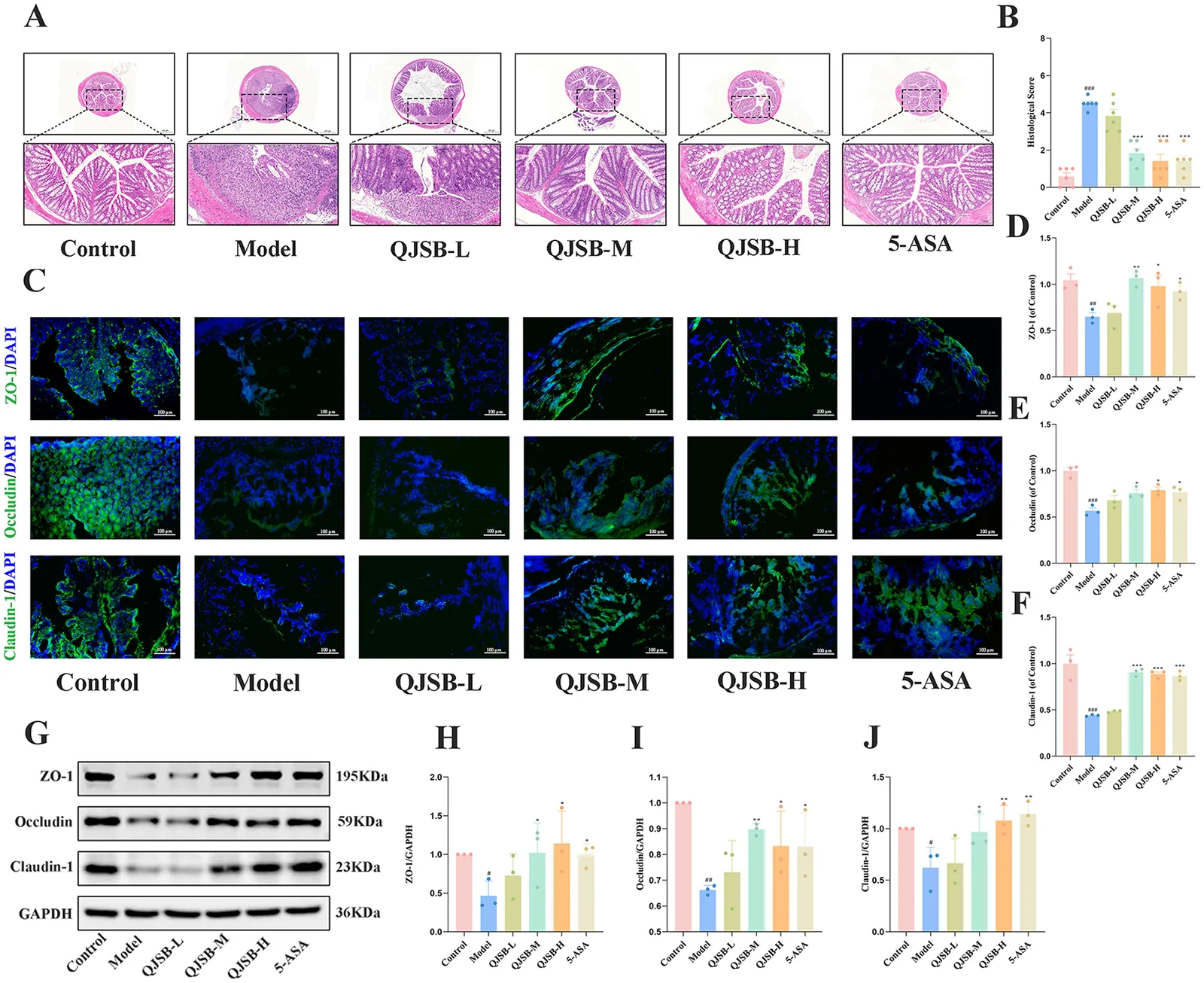
QJSB alleviates intestinal pathological damage in DSS-induced UC mice (*n* = 3). **(A)** colon tissue H&E staining (magnification 50 × and 200 × ); **(B)** histological scoring; **(C)** IF staining of ZO-1, Occludin and Claudin-1. Scale bar: 100 μm; **(D)**, **(E)** and **(F)** immunofluorescence quantification of Claudin-1, Occludin, and ZO-1; **(G)** Western blot analysis of ZO-1, Occludin, and Claudin-1; **(H)** Relative expression levels of ZO-1; **(I)** relative expression levels of Occludin; **(J)** relative expression levels of Claudin-1. (###*p* < 0.001, ##*p* < 0.01, #*p* < 0.05 vs. Control; *p < 0.05, **p < 0.01, ***p < 0.001 vs. Model).

### 3.6 QJSB alleviates inflammation in DSS-induced UC mice *in vivo*

To assess the anti-inflammatory properties of QJSB in the murine UC model, concentrations of key inflammatory markers were quantified in serum, colonic tissue, and fecal samples. Results showed significantly elevated MPO levels in the colon of the DSS model group (*p* < 0.05), while QJSB and 5-ASA treatment effectively reduced these levels (*p* < 0.05; Figure 6A). Relative to the control group, serum and colonic tissue concentrations of TNF-α, IL-1β, and IL-6 were markedly elevated in the DSS model group (*p* < 0.05). Administration of QJSB markedly inhibited the elevated levels of these inflammatory markers in mice with UC (*p* < 0.05), with the QJSB-M and QJSB-H groups exhibiting similar efficacy to 5-ASA (Figures 6B–G). Furthermore, *Bacteroides* is the primary gut microbiota producing lipopolysaccharide (LPS). Measurement of fecal LPS revealed increased levels in UC mice (*p* < 0.05), which were reduced following QJSB intervention (*p* < 0.05; Figure 6H). Moreover, both QJSB and 5-ASA treatment effectively normalized the upregulated protein expression of cyclooxygenase-2 (COX-2) and inducible nitric oxide synthase (iNOS; Figures 6I–K). We further analyzed correlations between key inflammatory factors and colonic metabolites using Spearman's algorithm. The results indicated strong positive correlations between the levels of arachidonic acid, methyl arachidonate, and prostaglandin H2 and the concentrations of LPS, MPO, IL-6, IL-1β, and TNF-α (*p* < 0.05; Figure 6L).

**Figure 6:**
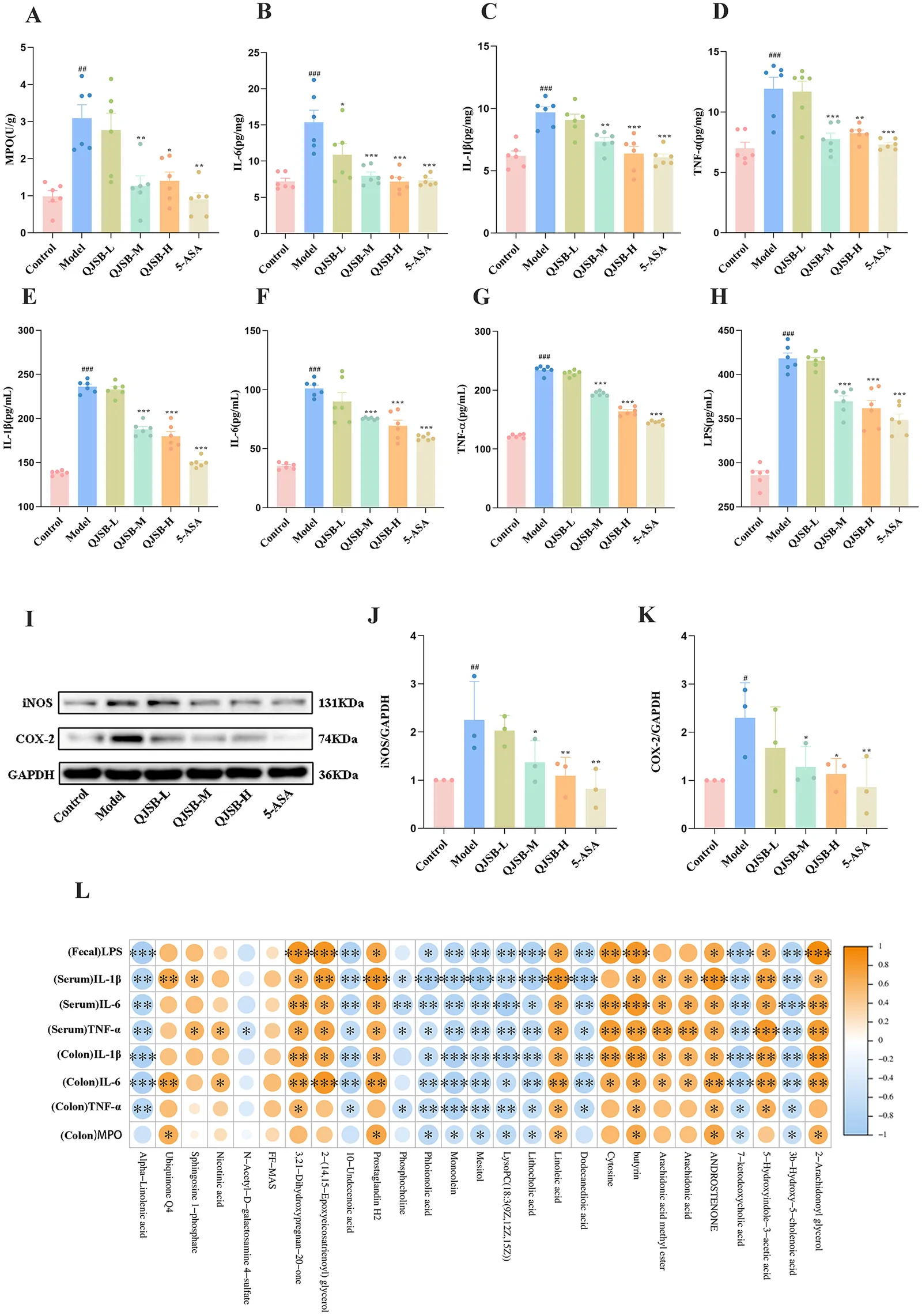
QJSB alleviates inflammation in DSS-induced UC mice *in vivo* (*n* = 6). **(A)**, **(B)**, **(C)** and **(D)** Levels of MPO, IL-6, IL-1β, and TNF-α in the colon; **(E)**, **(F)** and **(G)** serum levels of IL-1β, IL-6, and TNF-α; **(H)** levels of LPS in feces. **(I)** protein blots for iNOS and COX-2; **(J)** Relative expression levels of iNOS; **(K)** relative expression levels of COX-2; **(L)** correlation analysis between inflammatory mediators and colon-specific metabolites. (###*p* < 0.001, ##*p* < 0.01, #*p* vs. Control; *p < 0.05, **p < 0.01, ***p < 0.001vs. Model).

### 3.7 Network pharmacology analysis

We performed a network pharmacology analysis in this study. We obtained 642 putative targets of QJSB's bioactive constituents screened from the SwissTargetPrediction and TCMSP databases. In addition, we compiled 3,770 targets associated with UC from the GeneCards and OMIM resources. By intersecting these two sets of data, we identified 321 potential targets that QJSB may influence in the treatment of UC (Figure 7A). These 321 critical targets were further examined via the STRING platform, and a protein-protein interaction (PPI) network was constructed using Cytoscape 3.10.3 software, which led to the selection of 54 core targets (Figure 7B). The network depicting the relationship between the ten most active components, ranked by their degree, and the twenty intersecting gene targets is illustrated in Figure 7C. Through GO enrichment analysis, we found that at the biological process (BP) level, the pathways targeted by QJSB were significantly enriched in inflammatory response, positive regulation of canonical NF-κB signaling, arachidonate metabolic pathways, TLR4 signaling, and the MyD88-dependent TLR signaling pathway (Figure 7D). At the molecular function (MF) level, the highlighted pathways comprised NF-kappaB binding, integrin binding, prostaglandin E receptor activity, and arachidonate epoxygenase activity (Figure 7E). At the cellular component (CC) level, key components included cell surface, cell-cell junction, receptor complex, NF-kappaB p50/p65 complex, and plasma membrane (Figure 7F). KEGG pathway enrichment analysis (Figure 7G) revealed significantly affected pathways, including inflammatory bowel disease, the NF-κB signaling pathway, the TLR signaling pathway, arachidonic acid metabolism, and linoleic acid metabolism. Both GO enrichment and KEGG enrichment analyses suggest inflammation may be the core pathway through which QJSB exerts its therapeutic effects in UC.

**Figure 7:**
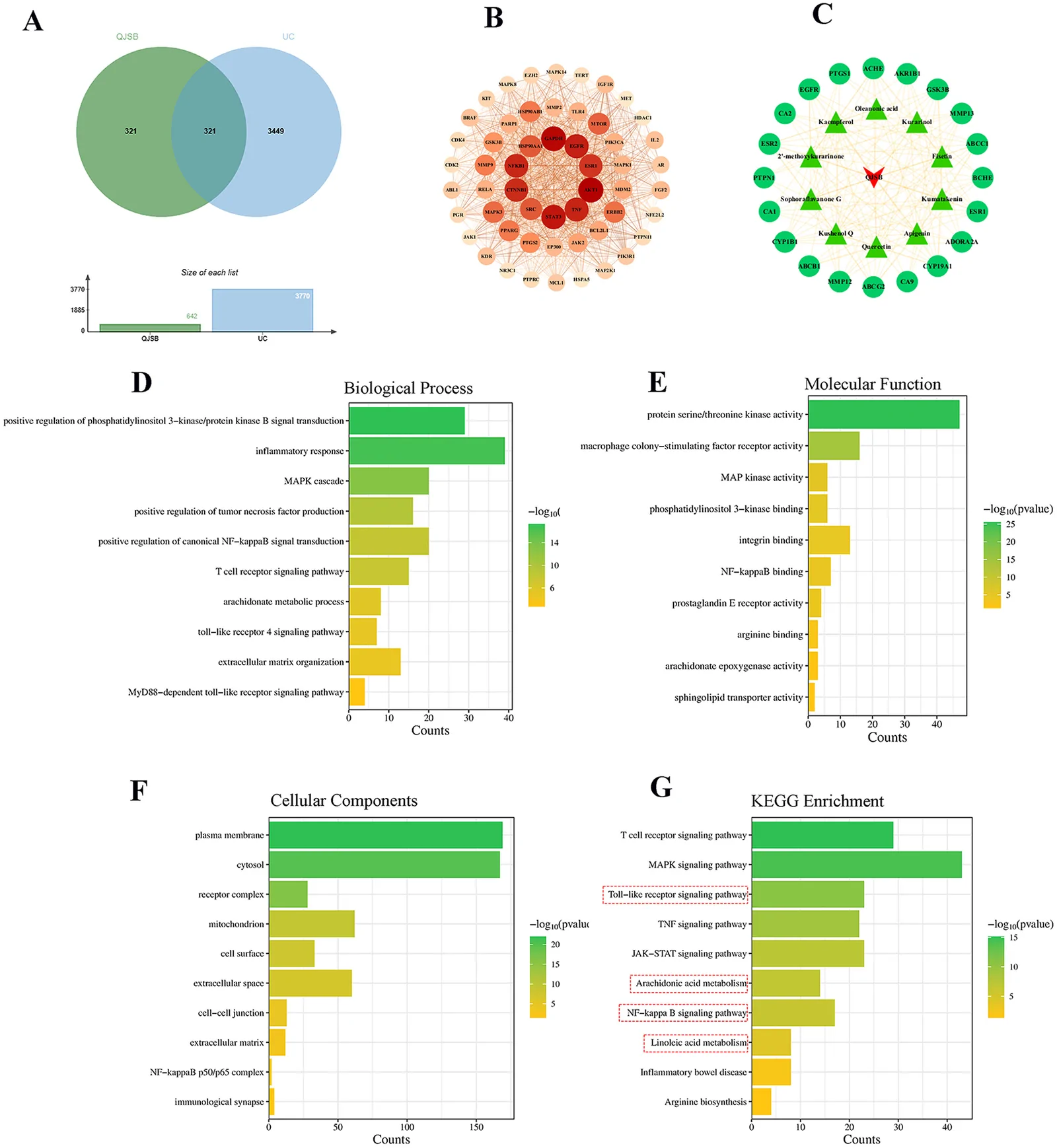
Network pharmacology study of QJSB treatment for mouse UC. **(A)** Venn diagram; **(B)** core target protein-protein interactions (PPIs); **(C)** “Drug-Component-Gene” network showing the intersection of the top 10 components by degree value and the top 20 genes; **(D)**, **(E)**, **(F)** GO enrichment analysis of QJSB-activated compounds targeting potential UC-related targets. BP, biological process; CC, cellular component; MF, molecular function; **(G)** KEGG enrichment analysis of QJSB-activated compounds targeting potential UC-related targets.

### 3.8 QJSB alleviates UC by blocking the TLR4/MyD88/NF-κB signaling pathway

Through KEGG pathway enrichment analysis in network pharmacology, we found that the Toll-like receptor and NF-κB pathways were significantly enriched, suggesting they may represent the action pathways through which QJSB intervenes in UC. Furthermore, we observed that QJSB downregulated LPS levels in the feces of UC mice. LPS is a key endogenous substance that activates the TLR4/MyD88/NF-κB pathway. Therefore, we speculate that QJSB may exert its effects through this signaling pathway. Western blot analysis revealed significant upregulation of TLR4 and MyD88 proteins, as well as increased p-IκBα/IκBα and p-p65/p65 ratios in colon tissues of DSS-treated mice (*p* < 0.05; Figures 8A–E), indicating DSS-induced activation of this signaling pathway. While intervention with QJSB-M and QJSB-H suppressed these protein expression levels (*p* < 0.05), approaching the effects of 5-ASA. Immunofluorescence staining results for p-IκBα and p-p65 were consistent with Western blot findings (Figures 8F–I) These results indicate that QJSB alleviates mouse UC by inhibiting the TLR4/MyD88/NF-κB pathway.

**Figure 8:**
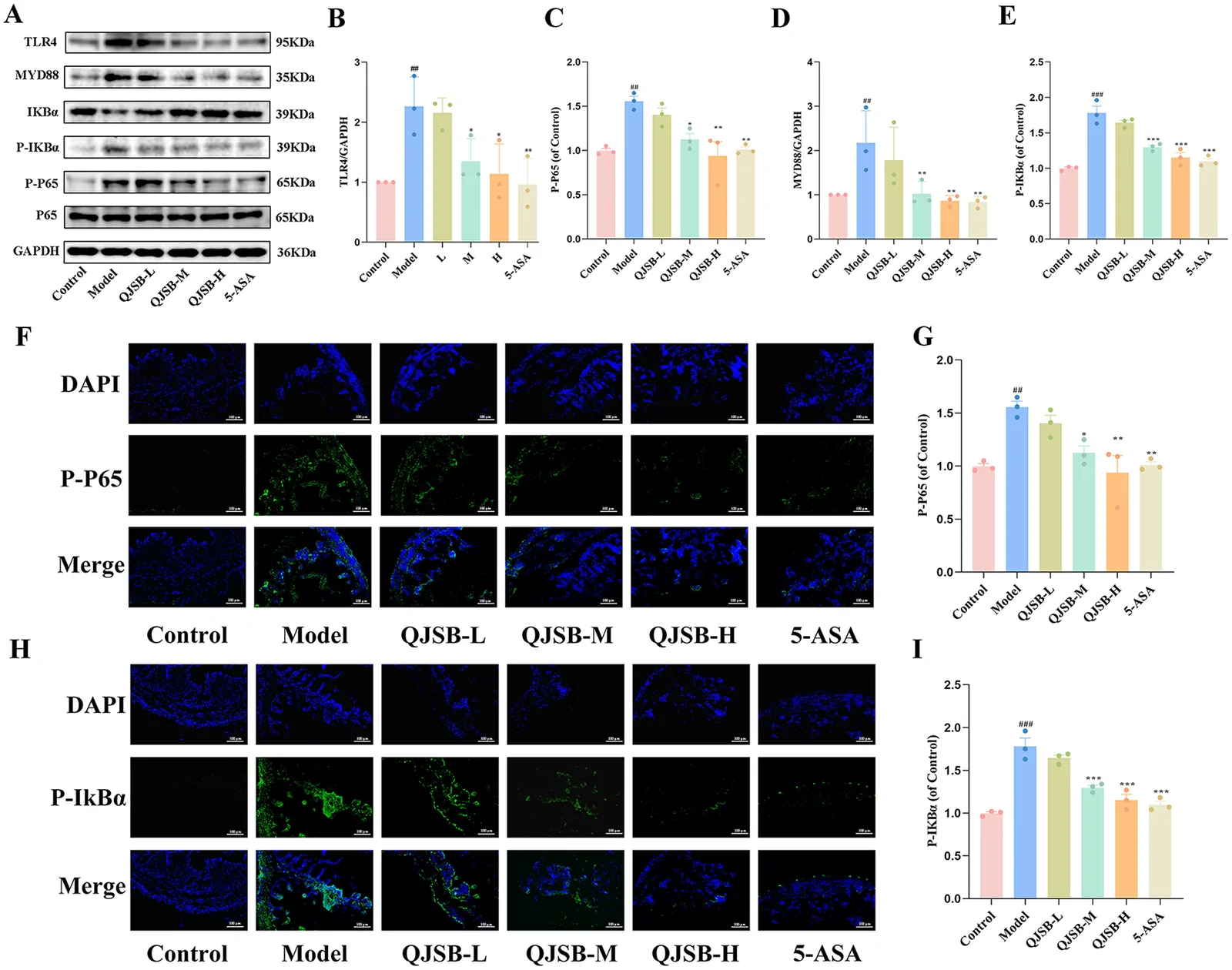
QJSB alleviates UC by blocking the TLR4/MyD88/NF-κB signaling pathway (*n* = 3). **(A)** Western Blot detection of TLR4/MyD88/NF-κB pathway protein expression in colon tissue; **(B)** relative expression levels of TLR4 protein; **(C)** relative expression levels of MyD88 protein; **(D)** relative expression levels of p-p65 protein; **(E)** relative expression levels of p-IκBα protein; **(F)** IF staining of p-p65. Scale bar: 100 μm; **(G)** immunofluorescence quantification of P-P65; **(H)** IF staining of p-IκBα. Scale bar: 100 μm; **(I)** immunofluorescence quantification of P-IkBα. (###*p* < 0.001, ##*p* < 0.01 vs. Control; *p < 0.05, **p < 0.01, ***p < 0.001 vs. Model).

## 4 Discussion

Characterized by symptoms such as abdominal pain, diarrhea, and rectal bleeding, ulcerative colitis (UC) represents a chronic form of inflammatory bowel disease (IBD). Its core pathological characteristics are intestinal barrier injury, persistent intestinal inflammation, and gut microbiota disturbance, which pose severe challenges to the effective treatment of this chronic disease (Liu et al., 2025). To date, anti-inflammatory or immunosuppressive drugs have remained the primary treatment approach for UC (Gros and Kaplan, 2023), but long-term use of such medications often leads to adverse reactions. Therefore, identifying safe, effective drugs without toxic side effects is crucial. Traditional Chinese medicine (TCM) has received growing recognition in recent years as a complementary treatment modality, showing promise in reducing intestinal inflammation and enhancing barrier restoration (Wang et al., 2022; Zhang et al., 2024a,b). QJSB, a classical TCM formula for treating leukopenia, has been demonstrated to modulate gut microbiota. In this study, we found that QJSB alleviates intestinal pathological damage in DSS-induced UC mice by reshaping the gut microbiota, improving metabolic disorders, and inhibiting the LPS-triggered activation of the TLR4/MyD88/NF-κB inflammatory signaling pathway.

Acting as a physical barrier, the intestinal epithelium blocks the entry of luminal bacteria and their endotoxins, thereby preventing potential invasion. The abnormal expression of tight junction proteins related to intestinal inflammation will damage the function of the intestinal epithelial barrier and can result in compromised barrier function (Guo et al., 2024; Wang et al., 2021). Tight junction proteins serve as fundamental structural elements essential for preserving the epithelial mucosal barrier's mechanical strength and regulating its permeability (Gong et al., 2022). Studies indicate that downregulation of intestinal tight junction proteins in UC mice may allow pathogens and toxins to breach the colonic barrier, disrupting mucosal barrier integrity and accelerating UC progression (Hu et al., 2022). In this study, QJSB increased the protein levels of ZO-1, occludin, and claudin-1, which promoted epithelial barrier integrity and alleviated UC.

A key contributor to the onset and progression of UC is gut microbiota dysbiosis, which is typically marked by a decline in beneficial bacterial populations, an expansion of pathogenic taxa, and a reduction in overall microbial diversity (Sultan et al., 2021; Yang and Li, 2022). This study confirmed via 16S rRNA sequencing that DSS-induced UC mice exhibited significantly reduced α-diversity in gut microbiota, enrichment of pathogenic bacteria (e.g., *Bacteroides*), and a concurrent reduction in beneficial bacteria (e.g., *Lactobacillus*)—findings consistent with the microbial profiles observed in clinical UC patients (Zuo et al., 2022). QJSB intervention not only restored gut microbiota abundance and diversity but also increased *Akkermansia* and *Lactobacillus* abundance while suppressing excessive *Bacteroides* proliferation. LEfSe analysis identified *Akkermansia* as the key dominant microflora mediating QJSB's protective effects. Existing research confirms that *Akkermansia muciniphila*, as a potentially beneficial microorganism, exerts positive effects on colitis, obesity, and related metabolic disorders (Zhang et al., 2024a,b; Bian et al., 2019; Cani et al., 2022); *Lactobacillus* prevents colitis in mice by augmenting tight junction protein abundance and preserving epithelial barrier integrity (Liu et al., 2011). Conversely, the genus *Bacteroides*, a major intestinal source of LPS, releases greater quantities of LPS when its levels rise, which subsequently promotes inflammatory pathway activation and compromises intestinal barrier integrity (Guo et al., 2015).

The gut microbiota and its metabolites play a significant role in the onset, progression, and regulation of UC (Zhang et al., 2026). Using non-targeted metabolomics, this study identified 27 and 56 differentially expressed metabolites in the colon and feces, primarily involving amino acid and fatty acid metabolism. At the level of amino acid metabolism, tryptophan is metabolized by the gut microbiota to produce microbiota-derived metabolites such as 5-hydroxyindoleacetic acid (5-HIAA) and indoxyl sulfate. 5-HIAA has been shown to possess anti-colitis activity; it restores intestinal epithelial barrier function by activating the aryl hydrocarbon receptor (AhR), upregulating IL-10, and inhibiting the NF-κB signaling pathway (Du et al., 2026). In this study, its levels were upregulated following QJSB intervention. In contrast, indoxyl sulfate levels are significantly elevated in IBD patients and are considered a marker of gut microbiota dysbiosis (Millet et al., 2023), in this study, these levels showed a decreasing trend following QJSB intervention. Furthermore, as an aromatic amino acid, excessive accumulation of phenylalanine can inhibit the activity of LPS detoxification-related phosphatases, exacerbating bacterial translocation and intestinal barrier damage (Chen et al., 2023), its levels were downregulated following QJSB intervention. At the level of fatty acid metabolism, linoleic acid and arachidonic acid metabolism have been shown to be key factors in regulating the pathological process of UC (Yang et al., 2025). Arachidonic acid can be metabolized by cyclooxygenase (COX) and lipoxygenase (LOX) into pro-inflammatory mediators such as prostaglandins and leukotriene B4 (Chachkovskyy et al., 2019). In UC mice, arachidonic acid and its downstream product, prostaglandin H_2_, were significantly elevated but decreased markedly following QJSB administration. Linoleic acid, a precursor to arachidonic acid, was significantly elevated in UC mice, and this trend was reversed following QJSB intervention. This study further verified through correlation analysis that *Akkermansia*, which is enriched by QJSB, shows a significant negative correlation with the pro-inflammatory metabolite arachidonic acid and a significant positive correlation with the anti-inflammatory metabolite 5-hydroxyindoleacetic acid. *Bacteroides*, on the other hand, exhibited the opposite trend.

Within the intricate pathophysiology of UC, chronic and uncontrolled inflammatory responses act as central drivers. Research confirms that the TLR4/MyD88/NF-κB pathway acts as a pivotal hub linking innate immune recognition, cytokine storms, and epithelial barrier damage (Chen et al., 2022; Kim et al., 2023). Within the intestinal microenvironment of UC patients, LPS produced by pathogenic bacteria such as *Bacteroides* continuously activates TLR4 (Shi et al., 2019). TLR4 primarily transmits signals by recruiting its downstream adaptor protein MyD88 (Facchini et al., 2020). MyD88 recruitment triggers the activation and nuclear translocation of the transcription factor NF-κB p65. Upon entering the nucleus, phospho-NF-κB p65 initiates the transcriptional expression of numerous proinflammatory cytokines, including TNF-α, IL-1β, and IL-6 (Yuan et al., 2023). In UC, excessive activation of this pathway leads to abnormally elevated levels of inflammatory mediators locally in the intestine and systemically throughout the body. This directly causes damage to the intestinal mucosal tissue, increased vascular permeability, and massive infiltration of immune cells (Bhat et al., 2022; Abraham et al., 2022). QJSB intervention lowered fecal LPS levels, inhibited the activation of the TLR4/MyD88/NF-κB pathway, and downregulated the expression of pro-inflammatory cytokines and inflammatory proteins. These results collectively confirm that QJSB exerts anti-UC effects by inhibiting the TLR4/MyD88/NF-κB signaling pathway.

The above results suggest that QJSB may exert its anti-UC effects by reshaping the gut microbiota composition, modulating microbiota-associated metabolites, and inhibiting the TLR4/Myd88/NF-κB inflammatory signaling pathway. However, the potential mechanisms by which QJSB intervenes in UC still require further experimental validation. It is essential to conduct future studies using fecal microbiota transplantation to verify the role of the microbiota, employ TLR4 agonists/antagonists to clarify pathway dependence, and utilize *in vitro* LPS-stimulation models to evaluate direct regulatory effects.

## 5 Conclusion

In summary, this study demonstrates that QJSB can effectively alleviate DSS-induced colitis in mice. The beneficial effects of QJSB on intestinal barrier damage and colonic inflammation may be related to its ability to regulate gut microbiota imbalance, correct metabolic disorders, and inhibit the TLR4/MyD88/NF-κB signaling pathway. These findings suggest that QJSB may exert its therapeutic effects on mice with UC through multiple synergistic mechanisms, providing experimental evidence for further research into its potential as a therapeutic candidate for UC.

## Data Availability

The original contributions presented in the study are included in the article/Supplementary material, further inquiries can be directed to the corresponding authors.
