# Supplementary material for: Qijiao Shengbai capsule ameliorates ulcerative colitis in mice by modulating gut microbiota and protecting the intestinal barrier, associated with suppression of the TLR4/MyD88/NF-κB pathway
Source: Front Microbiol. 2026 Sep 16;17:1875650. doi: 10.3389/fmicb.2026.1875650 (PMC13623905; doi:10.3389/fmicb.2026.1875650)
Supplement: Supplementary file 1 [file Supplementary_File_1.docx]

Supplementary Material

**Qijiao Shengbai capsule ameliorates ulcerative colitis in mice by modulating gut microbiota and protecting the intestinal barrier, associated with suppression of the TLR4/MyD88/NF-κB Pathway**

**Yi Lu^1,2†^, Min Zhang^1,2†^, Siyue Jiang^1,2^, Shuo Zhang^1,2,3^, Zhiyu Chen^1,2^, Jueben Wang^1,2,5^, Li Liu^1,2,5^, Pengjiao Wang^1,2^*, Xiuli Gao^1,2,4^***

^1^State Key Laboratory of Discovery and Utilization of Functional Components in Traditional Chinese Medicine & School of Pharmaceutical Sciences, Guizhou Medical University, Guiyang 561113, China, ^2^Engineering Research Center of Microbiology and Biochemical Pharmaceutical, Guizhou Provincial Department of Education, Guiyang 561113, China, ^3^Experimental Animal Center of Guizhou Medical University, Guiyang 561113, China, ^4^Guizhou Provincial Engineering Research Center of Food Nutrition and Health, Guizhou Medical University, Guiyang 561113, China, ^5^Guizhou Hanfang Pharmaceutical Co., Ltd., Guiyang 550014, China

Supplementary Tables

Supplementary Table 1. Disease activity index score (DAI score).

| Score | Weight loss | Stool consistency | Rectal bleeding |
| --- | --- | --- | --- |
| 0 | None | Normal | No bleeding |
| 1 | 1-5% | — | — |
| 2 | 5-10% | Loose stool | Mild bleeding |
| 3 | 10-15% | — | — |
| 4 | More than 15% | Watery diarrhea | Prominent bleeding |

Supplementary Table 2. Tentative identiﬁcation of potential active ingredients in QJSB extracts by UHPLC-ESI-Q-Exactive plus Orbitrap-MS.

| No. | RT/min | Tentative identification | Molecular Formular | [M+H]^+^ | [M+H]^-^ | Source |
| --- | --- | --- | --- | --- | --- | --- |
| 1 | 3.606 | Protocatechuic acid-3-0-glc | C_13_H_16_O_9_（+0.25） |  | 315.07278 | Xue Renshen |
| 2 | 2.091 | Theophylline | C_7_H_8_N_4_O_2_（+3.41） | 181.07262 |  | Xue Renshen |
| 3 | 7.606 | 2-hydroxy-4-methoxysalicylic acid | C_8_H_8_O_4_（-4.14） |  | 167.03423 | Xue Renshen |
| 4 | 8.941 | 7-Hydroxychromone | C_9_H_6_O_3_（-0.37） | 163.03926 |  | Xue Renshen |
| 5 | 11.61 | 3,7-dihydroxyflavanone | C_15_H_12_O_4_（+0.66） | 257.08127 |  | Xue Renshen |
| 6 | 9.275 | Schizandriside | C_25_H_32_O_10_(+2.06) | 491.19357 |  | Xue Renshen |
| 7 | 11.558 | 3,7,4'-trihydroxy-3'-methoxyflavanonol | C_16_H_14_O_6_(+2.2) | 303.08698 |  | Xue Renshen |
| 8 | 10.509 | 3,7,4',5'-tetrahydroxyflavone | C_15_H_10_O_6_(-0.39) | 287.05496 |  | Xue Renshen |
| 9 | 11.353 | Daidzein | C_15_H_10_O_4_(-0.08) | 255.06563 |  | Xue Renshen |
| 10 | 13.152 | Genistein | C_15_H_10_O_5_(0.95) | 269.04572 |  | Xue Renshen |
| 11 | 15.451 | Glucopyranose calycosin | C_16_H_12_O_5_(-2.81) | 285.07556 |  | Xue Renshen |
| 12 | 11.935 | Fisetin | C_15_H_10_O_6_(-0.62) |  | 285.04080 | Xue Renshen |
| 13 | 14.446 | Liquiritigenin | C_15_H_12_O_4_(-1.06) |  | 255.06590 | Xue Renshen |
| 14 | 12.401 | (-)-3,7,3'-trihydroxy-4'-methoxyflavan | C_16_H_16_O_5_(-1.99) | 289.10648 |  | Xue Renshen |
| 15 | 16.908 | Farnish | C_16_H_12_O_5_(-1.93) |  | 283.06113 | Xue Renshen |
| 16 | 9.042 | Coumestrol | C_15_H_8_O_5_(2.79) | 269.04572 |  | Xue Renshen |
| 17 | 13.177 | 4,4'-dihydroxy-2'-methoxychalcone | C_16_H_14_O_4_(-0.99) |  | 269.08203 | Xue Renshen |
| 18 | 12.971 | 3,7,3'-trihydroxyflavanonol | C_15_H_12_O_5_(0.05) |  | 271.06110 | Xue Renshen |
| 19 | 11.912 | 6,4'-dihydroxy-3'-methoxyaurone | C_16_H_12_O_5_(-2.24) | 285.07529 |  | Xue Renshen |
| 20 | 15.180 | 3,5,7'-trihydroxyflavanonol | C_15_H_12_O_5_(0.53) |  | 271.06119 | Xue Renshen |
| 21 | 15.750 | 7'-hydroxy-3',4'-dimethoxyisoflavone | C_17_H_14_O_5_(-0.31) | 299.09134 |  | Xue Renshen |
| 22 | 12.078 | 3'-hydroxy-2',4'-dimethoxy-isoflavan-6-0-glc | C_23_H_28_O_10_(+0.28) |  | 403.16125 | Xue Renshen |
| 23 | 14.664 | Formononetin | C_16_H_12_O_4_(-1.41) | 269.08057 |  | Xue Renshen |
| 24 | 15.998 | 7-hydroxy-4'-methoxyvflavanone | C_16_H_14_O_4_(0.61) | 271.09656 |  | Xue Renshen |
| 25 | 24.057 | Erycibenin D | C_26_H_30_O_6_(-1.2) | 439.21170 |  | Xue Renshen |
| 26 | 18.411 | Oleanonic acid | C_30_H_46_O_3_(-1.3) | 455.35159 |  | Da Zao |
| 27 | 1.447 | N-methylcytisine | C_12_H_16_N_2_O(1) | 205.13374 |  | Ku Shen |
| 28 | 1.535 | Cytisine | C_11_H_14_N_2_O(1.61) | 191.11818 |  | Ku Shen |
| 29 | 3.527 | Matrine | C_15_H_24_N_2_O(-1.66) | 249.19566 |  | Ku Shen |
| 30 | 4.587 | Sophoridine | C_15_H_24_N_2_O(-1.67) | 249.19582 |  | Ku Shen |
| 31 | 2.033 | Oxymatrine | C_15_H_24_N_2_O_2_(+0.98) | 265.19153 |  | Ku Shen |
| 32 | 6.726 | 9α-hydroxymatrine | C_15_H_24_N_2_O_2_(+2.49) | 265.19171 |  | Ku Shen |
| 33 | 8.891 | Sophoranol | C_15_H_24_N_2_O_2_(+2.49) | 265.19202 |  | Ku Shen |
| 34 | 2.997 | 9α-hydroxyoxysophocarpine | C_15_H_24_N_2_O_3_(+2.09) | 281.18619 |  | Ku Shen |
| 35 | 7.878 | 5α-hydroxysophocarpine | C_15_H_22_N_2_O_2_(+1.79) | 263.17587 |  | Ku Shen |
| 36 | 1.892 | 9α-hydroxysophocarpine | C_15_H_22_N_2_O_2_(+1.9) | 263.17575 |  | Ku Shen |
| 37 | 8.227 | 3'-hydroxy-4'-methoxy-isoflavone-7-O-api-O-glc | C_27_H_30_O_14_(+1.7) | 579.17267 |  | Ku Shen |
| 38 | 10.534 | 5-hydroxy-4'-methoxy-isoflavone-7-O-glc-O-api | C_27_H_30_O_14_(1.45) |  | 577.15588 | Ku Shen |
| 39 | 11.149 | 7-hydroxy-3'-methoxy-isofavone | C_16_H_12_O_4_(-0.76) | 269.08069 |  | Ku Shen |
| 40 | 11.935 | Kushenol H or Kushenol K | C_26_H_32_O_8_(-1.49) | 473.21713 |  | Ku Shen |
| 41 | 15.897 | Kushenol H isomer | C_26_H_32_O_8_(-1.38) | 473.21707 |  | Ku Shen |
| 42 | 17.517 | Kurarinol | C_26_H_32_O_7_(-0.14) | 457.22205 |  | Ku Shen |
| 43 | 19.09 | Kuraridinol | C_26_H_32_O_7_(-0.14) | 455.20749 |  | Ku Shen |
| 44 | 14.494 | 2'-hydroxy-isxanthohumol | C_21_H_22_O_6_(-0.44) |  | 369.13397 | Ku Shen |
| 45 | 18.764 | 8-isopentenyl-7,4'-dihydroxy-5-methoxy-flavanonol | C_21_H_22_O_6_(0.02) | 371014923 |  | Ku Shen |
| 46 | 14.070 | Sophorai soflavanone A | C_21_H_22_O_6_(-0.13) | 371.14905 |  | Ku Shen |
| 47 | 21.411 | Xanthohumol | C_21_H_22_O_5_(0.64) | 355.15417 |  | Ku Shen |
| 48 | 17.149 | Kushenol Q | C_25_H_30_O_7_(-0.94) |  | 441.19189 | Ku Shen |
| 49 | 18.486 | Kushenol Q isomer | C_25_H_30_O_7_(-0.58) |  | 441.19186 | Ku Shen |
| 50 | 20.018 | Kushenol L | C_25_H_28_O_7_(0.37) |  | 439.17633 | Ku Shen |
| 51 | 17.419 | Kurarinone | C_26_H_30_O_6_(-1.28) | 439.21155 |  | Ku Shen |
| 52 | 20.649 | Kurarinone isomer | C_26_H_30_O_6_(-1.26) | 439.21146 |  | Ku Shen |
| 53 | 17.395 | Desmethylxanthohumol | C_20_H_20_O_5_(-1.13) | 339.12344 |  | Ku Shen |
| 54 | 18.965 | Kushenol R | C_26_H_30_O_5_(0.31) | 423.21652 |  | Ku Shen |
| 55 | 21.765 | 8-lavandulyl Kaempferol | C_26_H_30_O_5_(0.26) | 423.21716 |  | Ku Shen |
| 56 | 17.125 | Kushend C | C_25_H_26_O_7_(0.08) | 439.17596 |  | Ku Shen |
| 57 | 19.304 | 2'-methoxykurarinone | C_27_H_32_O_6_(-0.55) | 453.22684 |  | Ku Shen |
| 58 | 19.522 | Norkurarinone or Sophoraflavanone G | C_25_H_28_O_6_(0.19) |  | 423.18167 | Ku Shen |
| 59 | 23.936 | Sophoraflavanone G | C_25_H_28_O_6_(0.13) |  | 423.18146 | Yin Yanghuo |
| 60 | 10.784 | Dihydrodemethylicaritin-7-0-di-glc | C_32_H_40_O_16_(-1.07) |  | 679.22363 | Yin Yanghuo |
| 61 | 3.071 | Quinic acid | C_7_H_12_O_6_(-3.44) |  | 191.005539 | Yin Yanghuo |
| 62 | 7.219 | Quinic acid isomer | C_7_H_12_O_6_(-2.81) |  | 191.05585 | Yin Yanghuo |
| 63 | 7.316 | Chlorogenic acid | C_16_H_18_O_9_(2.11) |  | 353.08844 | Yin Yanghuo |
| 64 | 6.366 | Neochlorogenic acid | C_16_H_18_O_9_(1.71) |  | 353.08868 | Yin Yanghuo |
| 65 | 7.948 | 3-coumaroylquinic acid | C_16_H_18_O_9_(1.71) |  | 337.09433 | Yin Yanghuo |
| 66 | 10.582 | Epimedin B isomer | C_38_H_48_O_19_(-0.97) |  | 807.27063 | Yin Yanghuo |
| 67 | 10.336 | Ikarisoside C | C_38_H_48_O_20_(-0.67) |  | 823.26581 | Yin Yanghuo |
| 68 | 10.436 | Epimedoside E | C_37_H_46_O_19_(-1.17) |  | 793.25586 | Yin Yanghuo |
| 69 | 10.082 | Icarisoside B | C_32_H_38_O_15_(-1.4) |  | 661.21326 | Yin Yanghuo |
| 70 | 10.835 | 3'-hydroxyicariine-3-O-rha-7-O-glc | C_39_H_50_O_20_(-1.93) |  | 837.28131 | Yin Yanghuo |
| 71 | 12.032 | icariin isomer | C_33_H_40_O_15_(-1.01) |  | 675.22882 | Yin Yanghuo |
| 72 | 14.590 | icariin | C_33_H_40_O_15_(-2.06) |  | 675.22870 | Yin Yanghuo |
| 73 | 15.775 | icariin isomer | C_33_H_40_O_15_(-1.85) |  | 675.22876 | Yin Yanghuo |
| 74 | 14.349 | Sagittatoside B | C_32_H_38_O_14_(-1.98) |  | 645.21802 | Yin Yanghuo |
| 75 | 12.177 | Sagittatoside B isomer | C_32_H_38_O_14_(-0.48) |  | 645.21857 | Yin Yanghuo |
| 76 | 13.943 | Demethy lanhydroicaritin-7-0-glc | C_26_H_28_O_11_(0.32) |  | 515.15564 | Yin Yanghuo |
| 77 | 12.376 | 2"-O-rhamnoshl icariside II | C_33_H_40_O_14_(-1.25) |  | 659.23370 | Yin Yanghuo |
| 78 | 16.317 | 2"-O-Orhamnoshl icariside II isomer | C_33_H_40_O_14_(-1.98) |  | 659.13318 | Yin Yanghuo |
| 79 | 17.003 | Baohuoside I | C_27_H_30_O_10_(-0.92) |  | 513.17615 | Yin Yanghuo |
| 80 | 8.891 | Calycosin-7-O-glc | C_22_H_22_O_10_(0.97) | 447.17615 |  | Huang Qi |
| 81 | 12.497 | Ferulic acid | C_10_H_10_O_4_(1.1) | 195.06540 |  | Huang Qi |
| 82 | 10.460 | isomucronulatol-4'-O-api | C_22_H_26_O_9_(-1.02) | 435.16455 |  | Huang Qi |
| 83 | 11.149 | Ononin | C_22_H_22_O_9_(-1.11) | 431.13364 |  | Huang Qi |
| 84 | 10.913 | Calycosin-7-0-glc(OAc) | C_24_H_24_O_11_(0.63) | 489.13919 |  | Huang Qi |
| 85 | 11.711 | Methylinissolin-3-O-glc | C_23_H_26_O_10_(-0.85) | 463.15973 |  | Huang Qi |
| 86 | 11.711 | 3-hydroxy-9,10-dimethoxy-pterocarpane | C_17_H_16_O_5_(-0.3) | 301.10712 |  | Huang Qi |
| 87 | 15.354 | 7,2'-dinhydroxy-3',4'-dimethoxyisoflavane | C_17_H_18_O_5_(-0.16) | 303.12265 |  | Huang Qi |
| 88 | 12.153 | Kumatakenin | C_17_H_14_O_6_(-0.11) | 315.08670 |  | Huang Qi |
| 89 | 15.002 | 10-hydroxy-3,9-dimethoxy-pterocarpane isomer | C_17_H_16_O_5_(-0.29) | 301.10724 |  | Huang Qi |
| 90 | 15.377 | Senkyunolide or isomer | C_12_H_14_O_3_(-3.12) |  | 205.08629 | Dang Gui |
| 91 | 14.664 | Butylidenephthalide isomer | C_12_H_12_O_2_(-1.07) |  | 169.09077 | Dang Gui |
| 92 | 17.759 | Ligustillide isomer | C_12_H_14_O_2_(-0.69) | 191.10684 |  | Dang Gui |
| 93 | 19.450 | E-butylidenephthalide | C_12_H_12_O_2_(-1.05) | 191.10658 |  | Dang Gui |

## Supplementary Figures

**Supplementary Figure 1.** Images of the colon.

**Supplementary Figure 2.** Permutation test. (A) Colon, (B) Feces.

**Supplementary Figure 3.** Alpha diversity. (A) Shannon Index, (B) ACE Index, (C) chao1 Index.
